# Understanding Public Perceptions of Normal Aging and Dementia in the United States

**DOI:** 10.1093/geroni/igaf122.4274

**Published:** 2025-12-31

**Authors:** Lucas Hamilton, Max Coleman, Anne Sprecher, Lauren Rutter

**Affiliations:** Augustana University, Sioux Falls, South Dakota, United States; The University of Utah, Salt Lake City, Utah, United States; Augustana University, Sioux Falls, South Dakota, United States; Indiana University Bloomington, Bloomington, Indiana, United States

## Abstract

The rising prevalence of Alzheimer’s disease and related dementia (ADRD) presents major public health concerns. Unfortunately, public knowledge about ADRD significantly diverges from scientific consensus. Misinformation and ageism have led to heightened stigma against people with ADRD. Characteristics of the stigmatized (i.e., people with ADRD) and of the general public both influence stigma; however, this evidence mainly comes from international samples. Building on this evidence, this study addresses ADRD stigma in the United States using stratified quota sampling of 1,115 adults to match national demographics. Using the well-established vignette approach, a 2 (Gender: Male, Female) × 3 (Cognitive Status: Normal, MCI, ADRD) between-subjects design was employed. Symptoms of the vignette character mirror the cognitive domains affected by ADRD and were reviewed by geriatric neuropsychologists to ensure accuracy. Stigma was shaped by individual differences more than vignette factors. Using multiple regression analyses, higher stigma was associated with less knowledge, younger age, being male, non-White race, identifying as a Republican, and living in areas with higher ADRD prevalence. Surprisingly, personal contact was not associated with less stigma. Only a third of participants (35.8%) correctly identified normal cognitive aging, and a sixth (16.8%) mislabeled impairment as normal. Scores on the Dementia Knowledge Assessment scale were roughly 50% with only 77 respondents (6.9%) correctly answering 20 items or more, which is the baseline seen in some international samples. Therefore, efforts to reduce ADRD stigma need to be tailored to address informational deficits and narratives that perpetuate negative perceptions in certain groups.

